# Retraction: Experimental study on the properties of ultra-high-strength geopolymer concrete with polypropylene fibers and nano-silica

**DOI:** 10.1371/journal.pone.0346527

**Published:** 2026-04-06

**Authors:** The PLOS One Editors

The *PLOS One* Editors retract this article [1] due to concerns about:

- Compliance with PLOS policies on Authorship.
- Reliance on retracted work cited in References 8, 10, 14, 17, 34, and 37.

RMG and JdPG agreed with the retraction. FA, OZ, SA, and MMA either did not respond directly or could not be reached.
